# Incorporation of a Cost of Deliberation Time in Perceptual Decision Making

**DOI:** 10.1523/JNEUROSCI.2426-24.2025

**Published:** 2025-07-07

**Authors:** Shinichiro Kira, Ariel Zylberberg, Michael N. Shadlen

**Affiliations:** ^1^Department of Neurobiology, Harvard Medical School, Boston, Massachusetts 02115; ^2^Zuckerman Mind Brain Behavior Institute, Columbia University, New York, New York 10027; ^3^Virtual Confidence and Metacognition Laboratory, Rochester, New York 14618; ^4^Kavli Institute for Brain Science, Columbia University, New York, New York 10027; ^5^Grossman Center for the Statistics of the Mind, Columbia University, New York, New York 10027; ^6^Howard Hughes Medical Institute, Columbia University, New York, New York 10027

**Keywords:** collapsing decision bounds, decision making, drift-diffusion model, random-dot motion task, speed-accuracy tradeoff, time costs

## Abstract

Many decisions benefit from the accumulation of evidence obtained sequentially over time. In such circumstances, the decision-maker must balance speed against accuracy, while considering the cost associated with the passage of time. A neural mechanism to achieve this balance is to accumulate evidence and to terminate the deliberation when enough evidence has accrued. To accommodate time costs, it has been hypothesized that the criterion to terminate a decision may become lax as a function of time. Here we tested this hypothesis by manipulating the cost of time in a perceptual choice–reaction time (RT) task. Human participants (both sexes) discriminated the direction of motion in a dynamic random-dot display, which varied in difficulty across trials. Unbeknownst to the participants, halfway through the experiment, we increased the time pressure by canceling a small fraction of trials, mimicking a broken fixation, if they had not made a decision by a provisional deadline. This subtle manipulation led participants to make faster but less accurate decisions. Choice and RT were well explained by a bounded evidence-accumulation process. We developed a novel computational method to estimate the time-dependent changes in the stopping bounds directly from the participants’ RT and choice data. Our analysis revealed that the bounds decline as a function of time, and that this decline is steeper following the time–cost manipulation. The time-varying decision bounds approximate an optimal stopping policy, although the specific bound shape is idiosyncratic across individuals.

## Significance Statement

This study demonstrates that human decision-makers flexibly adjust the balance between decision speed and accuracy in response to subtle changes in the cost of deliberation time. This adjustment is achieved by modulating decision criteria—that is, the amount of evidence required before committing to a choice. Using a novel computational approach, the authors show that these criteria decline over time within a trial, and do so more rapidly when the cost of deliberation is higher. The time-varying decision criteria approximate an optimal stopping policy, although the specific bound shape is idiosyncratic across individuals. These findings provide new insight into the computational mechanisms that support adaptive decision making.

## Introduction

A decision is a commitment to a proposition or plan based on evidence from the environment or memory. Often a decision-maker is allowed the opportunity to deliberate over a sequence of samples of evidence, which bear on the proposition at hand. In that case, the decision is not only just about the proposition but also about when to stop deliberating. Such sequential sampling with optional stopping invites a policy that would balance the speed of making a decision against accuracy. For decisions about a perceptual category (e.g., present/absent or left/right), sequential sampling can be viewed as a natural extension of signal detection theory (Green and Swets, 1966; Luce, 1986; Gold and Shadlen, 2007; Shadlen and Kiani, 2013). In this theory, a criterion on evidence is viewed as a policy that instantiates a negotiation between the value (or cost) of answering one way or another, correctly or incorrectly. The same can be said of sequential sampling, with the addition that time, or the number of samples, is also part of the negotiation.

For many decisions, it is reasonable to presume that samples of evidence are conditionally independent and identically distributed. That is, given that the stimulus is in a particular state (e.g., present or absent; left or right), the samples of evidence are like random draws from some distribution of numbers. The samples are informative to the extent that the distributions associated with each hypothesis, state, or alternative are distinct. In that case, a sensible strategy is to accumulate evidence in units of the logarithm of the likelihood ratio (logLR) that the evidence would have been observed under one hypothesis or its alternative(s) (Wald, 1947; Wald and Wolfowitz, 1948; Laming, 1968; Link, 1992). The deliberation terminates when the logLR equals (or exceeds) a criterion level or bound. An important insight from signal detection theory is that the evidence need not be in units of logLR, per se; any monotonic transformation, such as conversion to spike rate, is sufficient to allow a single criterion to achieve the “negotiation” mentioned above (Green and Swets, 1966; Kira et al., 2015). However, the matter is more complex when the reliability of the evidence bearing on the decision is unknown.

If decisions are to be made about stimuli that differ in difficulty, as is often the case in tests of perception, the bound may need to be adjusted for each type of stimulus. More importantly, when costs are incurred as a function of decision time (e.g., to maximize reward rate), it may be sensible for the terminating bound to be time dependent (Gershman et al., 2015). In particular, it has been shown that if the level of difficulty is not known to the decision-maker at the beginning of deliberation, then in order to maximize the reward rate, the magnitude of the stopping bound should decline as a function of time (Frazier and Yu, 2008; Drugowitsch et al., 2012). We refer to this decline as a collapse of the decision bounds. There is experimental evidence for an equivalent mechanism shown in neurophysiological studies of perceptual decision making in monkeys (Hanks et al., 2014). While collapsing bounds are normative, it is unclear whether human decision-makers can implement such a strategy flexibly in response to temporal costs.

We wished to ascertain whether human participants would adjust their termination criteria to accommodate a subtle manipulation of the cost of deliberation time. We used a variant of a direction discrimination task for which it is well established that choices and reaction times (RTs) conform to the sequential sampling models. We developed a novel computational approach to estimate the time-course of decision-termination bounds from participants’ choices and RTs. The analysis revealed that each participant exhibited an idiosyncratic collapsing bound profile, achieving near-optimal performance. When time costs were subtly increased halfway through the experiment, participants adjusted their speed–accuracy tradeoff. These adjustments were largely driven by changes in the initial height and the rate of collapse of the bounds, while preserving each participant’s distinct profile.

## Materials and Methods

### Participants

Participants were four young adults (three males and one female) with normal or corrected-to-normal visual acuity. All the participants were involved in the experiment with informed written consent. They either volunteered or were paid $15/h during the experiment. All experimental procedures were approved by the institutional review board at the University of Washington.

### Apparatus

Participants were seated in front of the display. A chin rest and forehead bar were used to stabilize the head of the participant for the duration of each experimental session. The stimuli were displayed on a flat-screen cathode ray tube video monitor (19-in. View Sonic PF790, 800 by 600 pixels, viewing distance = 57 cm, subtending 35.0° by 26.6° of visual angle with 22.1 pixel/deg at screen center; refresh rate = 75 Hz) controlled by a Macintosh G5 (2× 2.8 GHz, Mac OS 10.5 with an ATI Radeon HD 2,600 graphics card). Stimuli were generated using the Psychophysics Toolbox Version 2 (Brainard, 1997; Pelli, 1997) for MATLAB (Version 7.5, Mathworks). Eye movements in one eye were recorded with a 1,000 Hz sampling rate by a noninvasive infrared video-tracking system (EyeLink 1,000, SR Research).

### Motion direction discrimination task

**Figure 1.**
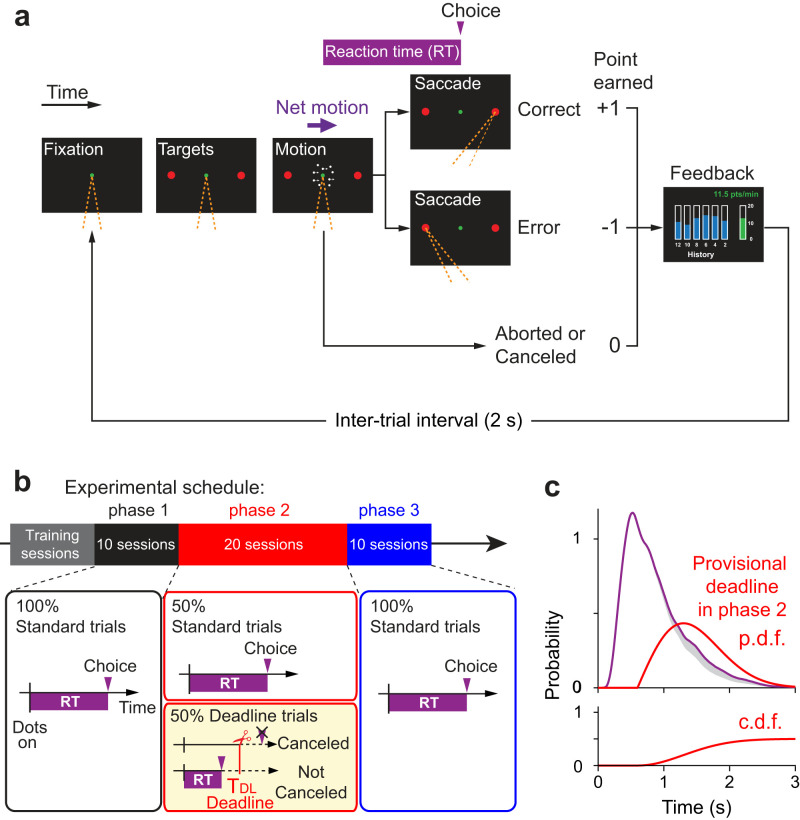
Experimental design. Participants made perceptual decisions in a choice–RT design. Unbeknownst to the participants, the sessions were organized in three phases. ***a***, Random-dot motion (RDM) task. Participants initiate a trial by fixating a central spot (green). Two peripheral choice targets then appear (red circles). After a variable delay, the RDM appears in a virtual aperture centered on the fixation point. Participants must decide the net direction of motion (right or left) and report this decision by making an eye movement to the choice-target in the corresponding direction. Participants earn 1 point for a correct decision and lose 1 point for an incorrect decision. The participant receives immediate auditory feedback for correct and erroneous choices as well as visual feedback about their points earning rate over the preceding 2 min (green bar) and its history during the session (blue bars). Trials aborted by poor eye fixation or canceled by provisional deadlines (phase 2 only) incur no change in points but affect the points earning rate. Orange dashed lines indicate the gaze direction. ***b***, Experimental schedule. The participants performed training sessions until their point-earning rate was stable, followed by the three phases of the experiment. Phase 1 comprised 10 sessions identical to the training. Phase 2 comprised 20 sessions in which half of trials (randomly interleaved) were assigned provisional deadlines (yellow box). Phase 3 comprised 10 sessions identical to phase 1 (i.e., without provisional deadlines). ***c***, *Top*, RT distribution from phase 1 (purple) and the distribution of provisional deadlines planned for phase 2 (red) for one participant (participant A). The RT distribution was smoothed by Epanechnikov kernel (inverted parabola) with SD = 0.1 s. *Bottom*, cumulative probability density function (cdf) of provisional deadlines. The deadlines are planned surreptitiously on half the trials, hence the area under the probability density function—and upper asymptote of the cdf—is 0.5. Shaded gray area shows ∼10% of RT distribution expected to be canceled by deadlines without a change in decision strategy from phase 1 to phase 2.

Participants discriminated the direction of dynamic random-dot motion in a choice–RT paradigm (Fig. 1*a*). Trials began with the appearance of a fixation spot (0.3° diameter) in the center of the screen. Participants were required to look at the fixation spot and maintain their fixation within 4° × 4° of their initial fixation. Once fixation was attained, two red targets (0.5° diameter) were presented 6° to the left and right of the fixation spot. After a variable delay (200–500 ms, exponentially distributed; mean 300 ms), the random-dot motion stimulus appeared within a 5° circular aperture centered on the fixation spot. Dots were 2 by 2 pixels (0.09° square), with a density of 16.7 dots/deg^2^/s. On each trial, the motion coherence was selected pseudorandomly from the list (±0, ±3.2, ±6.4, ±12.8, ±25.6, ±51.2%). The absolute value of the motion coherence—referred to as the *motion strength*—specifies the fraction of dots that are displaced in motion as opposed to randomly repositioned. The sign of the motion coherence indicates the direction of motion (negative for leftward and positive for rightward). The displacement of the coherently moving dots was such that the apparent speed of motion was five degrees visual angle per second. Details on the generation of the random-dot motion have been described in previous studies (Roitman and Shadlen, 2002; Palmer et al., 2005).

After the motion stimulus onset, participants were required to decide the direction of the motion stimulus and make a saccadic eye movement to the corresponding choice targets. When a saccade was detected within a ±3° rectangle around the chosen target, the trial was classified as either correct or error based on whether a chosen target direction and an assigned motion direction were the same or opposite, respectively. In these trials, RT was defined as the time from the motion stimulus onset to the initiation of the saccade, which was detected when the gaze first exited the 4° × 4° fixation window. When participants broke a fixation prematurely (e.g., eye blinks) or made an invalid saccade, these trials were terminated immediately and labeled as “aborted.”

Each trial was followed by a 2 s inter-trial interval. Participants performed trials repeatedly during a 13 min session (234.3 ± 11.0 trials/session, mean ± SD, *n* = 40 sessions per participant) and completed two sessions a day with a short break (5–10 min) in between.

### Scoring system

Participants earned one point for a correct choice and lost one point for an erroneous choice. The designation of correct or error was random for the 0% coherence trials. The total points did not change for aborted trials of any type (blinks, fixation inaccuracy, etc.). During the 2 s of an inter-trial interval, participants received visual and auditory feedback about their performance. The visual feedback indicated the total points in digits and the earned points per minute (points earning rate) in a bar graph, which included the current points earning rate during the latest 2 min and a history of the points earning rate for every 2 min from the beginning of a session (Fig. 1*a*). The participants were instructed to maximize their points earning rate during a session.

### Experimental schedule

We divided the experiment into three phases (Fig. 1*b*). We did not explain the experimental schedule to the participants, so that the prior knowledge of the task would not bias their behavior.

In phase 1, we measured the baseline performance. The participants were allowed to make a decision whenever they were ready within 5 s from the motion stimulus onset. These trials are referred to as *standard trials*. Because participants never experienced the maximum stimulus duration, participants performed the task as if there were no time limit. While the participants performed the task, we monitored their performance by tracking the total number of points earned and the earning rate (points per min). The participants performed the task repeatedly until their performance was deemed stable in terms of RT and points earning rate for at least 10 sessions. We use the last 10 sessions (2,154–2,389 trials) to characterize the baseline level of performance.

In phase 2 (20 sessions), we evaluated the effect of increased time cost by introducing provisional deadlines. The deadline trials and standard trials were randomly interleaved during this phase. If the participant made a decision before a deadline, the trial ended in the same way as in the standard trial. If participants had not made a decision by this deadline, however, the trial was terminated immediately as if they broke a fixation, and labeled as “canceled.” Each provisional deadline was randomly drawn from a time-shifted Rayleigh distribution:

$$P_{\mathrm{dl}}(t)=\left\{ \begin{array}{cc} \frac{t-t_{0}}{\sigma_{\mathrm{dl}}^{2}}\exp\;\left(-\frac{{\left(t-t_{0}\right)}^{2}}{2\sigma_{\mathrm{dl}}^{2}}\right)\; & \text{if }t>t_{0},\\ 0 & \text{otherwise, } \end{array}\right.\;(1)$$

where *t*_0_ is a time shift that sets the minimum time for the deadline, and *σ*_dl_ is the scale parameter of the distribution. These parameters were tailored for each participant based on his or her RT distributions measured in phase 1. Specifically, the parameters were chosen to cancel ∼10% of the trials in phase 2, where the participant maintains the same policy (i.e., decision speed and accuracy) as in phase 1. Note that the mean RT for deadline trials is expected to be shorter than RT for standard trials because RT has to be shorter than a deadline in each trial. To eliminate the confound that such attrition accounts for the shorter RT in phase 2 than in phase 1, deadline trials were excluded from all analyzes except the one devoted to the effect of cancelations on future RT (Fig. 9*b*,*c*).

In phase 3 (10 sessions), we evaluated a washout effect by eliminating the provisional deadlines. The participants performed standard trials as in phase 1.

### Statistical analysis of RT and choice data

We performed a *t*-test on *z*-scored RTs to evaluate the statistical significance of differences in RTs across experimental phases. For the comparison between two phases, RTs from both phases were combined for each signed motion coherence and *z*-scored. The *z*-scored RTs were then aggregated across motion coherences and compared between the two phases.

We used linear regression to test whether the change in RT from phase 1 to phase 2 could be explained by a steady decrease in RTs across sessions:

$$\text{RT}=\sum_{\left|\mathrm{coh}\right|}\beta_{\left|\mathrm{coh}\right|}I_{\left|\mathrm{coh}\right|}+\beta_{\mathrm{session}}k_{\mathrm{session}}+\beta_{\mathrm{phase}}I_{\mathrm{phase}}+\beta_{\mathrm{phase}\;\times \;\mathrm{session}}k_{\mathrm{session}}I_{\mathrm{phase}},\;(2)$$

where the *β* are fitted coefficients. $I_{\left|\mathrm{coh}\right|}$ is an indicator variable for the motion strength, which takes 1 if |coh|∈{0,3.2,6.4,12.8,25.6,51.2%} matches the motion strength of a given trial and 0 otherwise. *k*_session_ is an index for session number, where *k*_session_ = { − 10, …, − 1} for sessions in phase 1 and *k*_session_ = {0, …, 19} for phase 2. *I*_phase_ is an indicator variable that takes 1 for phase 2 trials and 0 otherwise. The regression analysis includes only standard trials (i.e., without provisional deadlines) from phases 1 and 2. We used a *z*-test to evaluate the null hypothesis $H_{0}\;:\;\beta_{\mathrm{phase}}=0$.

We used logistic regression to determine, for each subject, if there is a significant difference in accuracy between phase 1 and phase 2. The model that was fit separately for each participant is

$$\mathrm{logit}[p_{\mathrm{correct}}]=\beta_{\mathrm{coh}}|\mathrm{coh}|+\beta_{\mathrm{phase}}I_{\mathrm{phase}}|\mathrm{coh}|,\;(3)$$

where *I*_phase_ is an indicator variable that equals 1 for phase 2 trials and 0 otherwise. The parameter *β*_coh_ represents the effect of motion strength on the probability of a correct response, irrespective of phase, while *β*_phase_ quantifies how this effect differs for phase 2 trials. We evaluate the null hypothesis $H_{0}\;:\;\beta_{\mathrm{phase}}=0$, *z*-test.

We fit a similar model with data combined across participants:

$$\mathrm{logit}[p_{\mathrm{correct}}]=\sum_{s=1}^{4}\beta_{\mathrm{coh},\;s}I_{s}|\mathrm{coh}|+\beta_{\mathrm{phase}}I_{\mathrm{phase}}|\mathrm{coh}|,\;(4)$$

where *I*_s_ is an indicator variable that takes a value of 1 for trials completed by participant s and zero otherwise.

### Bounded drift-diffusion model

We analyzed participants’ performance in the framework of a bounded drift-diffusion model (DDM). In this model, the decision variable (DV), denoted by *x*, evolves over time by following a Wiener process with a constant drift:

$$\Delta x=\mu_{d}\Delta t+\xi \sqrt{\Delta t},\;(5)$$

where *ξ* is a random variable drawn from a standard normal distribution.

The drift term *μ*_*d*_ is proportional to the motion strength: $\mu_{d}=\kappa \cdot \mathrm{coh}$, where coh is the motion coherence with a positive or negative sign for rightward or leftward motion, respectively. *κ* is a free parameter that determines a signal-to-noise ratio in our model. Together, when the motion coherence is rightward (or leftward), it is likely to furnish positive (or negative) momentary evidence. The stochastic process produces the DV, *x*(*t*), which continues to evolve so long as −*B*(*t*) < *x*(*t*) < *B*(*t*), where ±*B*(*t*) are the upper and lower terminating bounds for right and left choices, respectively. Note that these bounds are symmetric about *x* = 0, but they are not assumed to be time-invariant (i.e., “flat”). The first passage time determines the choice and the decision time (*T*_d_). The RT includes additional sensory and motor delays, referred to as the *non-decision time* (*T*_nd_). We assume that *T*_nd_ is normally distributed with mean *μ*_nd_ and variance $\sigma_{\mathrm{nd}}^{2}$. The next section describes how we infer the shape of the decision bounds, *B*(*t*), without assuming a specific functional form.

### Nonparametric-bound drift-diffusion model

We developed a novel method to estimate the shape of the decision-termination bounds without assuming a specific functional form. The key intuition is that in the bounded DDM, if the drift rate is known, then the distribution of decision times is uniquely determined by the time-course of the bounds, ±*B*(*t*). Given this unique relation, the process can be inverted to infer *B*(*t*) from the empirical distribution of decision times.

Because of the non-decision latencies, the distribution of decision times is not directly observed; yet, we can approximate it from the observed RTs. The decision time for each trial *i* was estimated by $T_{d}^{\left(i\right)}=RT^{\left(i\right)}-\mu_{\mathrm{nd}}$, where *μ*_nd_ is the mean non-decision time. That is, we effectively ignore $\sigma_{\mathrm{nd}}^{2}$ when inferring the decision times from the observed RTs—a necessary simplification as we lack trial-specific information about the noise affecting non-decision time. The probability density function for the decision times, $p_{T_{d}}(t)$, was calculated from the single-trial decision times, $T_{d}^{\left(i\right)}$, using nonparametric kernel smoothing with an Epanechnikov kernel (inverted parabola with SD = 0.1 s).

After estimating $p_{T_{d}}(t)$, we alternated two operations, *propagation* and *match*, iteratively over time (Fig. 3*c*,*d*).

#### Propagation

In this step, we evolve the state of the DV for a single time step, from *t* to *t*′ = *t* + *dt*, by numerically solving the Fokker–Planck (FP) equation that describes the dynamics of a Wiener process with a constant drift:

$$\frac{\partial u}{\partial t}=\frac{\partial }{\partial x}\left[B\cdot u+D\cdot \frac{\partial u}{\partial x}\right],\;(6)$$

where $B=-\mu_{d}(\equiv -\kappa \cdot \mathrm{coh})$, *D* = *σ*^2^/2, and u(x,t)=p(x,t|coh) is the single-particle distribution function in the space 〈*x*, *t*〉. As indicated by the conditionalization, each motion coherence is treated independently (Fig. 3*c*). For numerical stability, we used a fully implicit scheme (Chang and Cooper, 1970) to numerically solve the FP equation.

For a single time step, we propagate the probability mass of the *unabsorbed* DV—that is the portion of p(x,t|coh) that lies between −*B*(*t*) and +*B*(*t*). Note that, however, the probability mass obtained after the propagation, $p(x,t^{\prime }|\mathrm{coh})$, is not restricted to lie between these bounds.

#### Match

If the bound heights at time *t*′ were known, then one could compute the total probability mass that is absorbed at time *t*′, *p*_abs_(*t*′), as

$$p_{\mathrm{abs}}(t^{\prime })=\sum_{i}p_{\mathrm{abs}}(t^{\prime }|{\mathrm{coh}}_{i})\cdot p({\mathrm{coh}}_{i}),\;(7)$$

where

$$p_{\mathrm{abs}}(t^{\prime }|{\mathrm{coh}}_{i})=\int_{-\infty }^{-B(t^{\prime })}p(x,t^{\prime }|{\mathrm{coh}}_{i})dx+\int_{B(t^{\prime })}^{\infty }p(x,t^{\prime }|{\mathrm{coh}}_{i})dx.\;(8)$$

In the *match* step, we find the value of ±*B*(*t*′) such that the probability mass that is absorbed at time *t*′, *p*_abs_(*t*′), is equal to the probability of terminating the decision at time *t*′ that is obtained from the empirical distribution of decision times, $p_{T_{d}}(t)$ (Fig. 3*d*). Since there is a monotonic relation between the bound height and the probability mass that is absorbed at the bound (Eq. 8), we can find the value of the symmetric bounds ±*B*(*t*′) by root finding, bracketing *B*(*t*′) within a sequence of diminishing intervals. Note that the empirical and model-derived absorption probability are matched after combining data across coherences (i.e., not per coherence level; Fig. 3*d*).

The *propagation* and *match* steps are repeated iteratively over time in increments of dt = 0.5 ms.

#### Likelihood of RT and choice

From the process described above, we obtain the joint distribution of decision time and choice for each motion coherence, $P(T_{d},\mathrm{choice}|\theta ,\mathrm{coh})$. We assume that non-decision time, *T*_nd_, is independent of decision time, motion strength, and direction, and model it as a normally distributed variable: $T_{\mathrm{nd}}∼N(\mu_{\mathrm{nd}},\sigma_{\mathrm{nd}}^{2})$. Consequently, the RT distribution is obtained by convolving the decision time distribution with the normally distributed non-decision times.

#### Fitting the model’s free parameters

We estimated the model’s free parameters, $\theta =[\kappa ,\mu_{\mathrm{nd}},\sigma_{\mathrm{nd}}]$, by maximum likelihood. For the experimental data, *D*, comprising RTs and choice directions across individual trials in each phase, we searched for a set of parameters that maximizes the likelihood of observing the data:

$$\underset{\theta }{argmax}\;P(D|\theta )=\underset{k}{\Pi }\;P({\mathrm{RT}}^{\left(k\right)},{\mathrm{choice}}^{\left(k\right)}|\theta ,{\mathrm{coh}}^{\left(k\right)}),\;(9)$$

where *k* indexes individual trials for a specific participant and phase.

To estimate the model parameters *θ*, we minimize the negative log-likelihood of the observed choice and RT data using the simplex search method with boundary constraints (D’Errico, 2025). The optimization begins with an initial guess, *θ*_initial_, drawn uniformly from the following ranges: *κ* ∈ [5, 40], *μ*_nd_ ∈ [0.01, 0.6], and *σ*_nd_ ∈ [0.001, 0.08]. We use nonparametric-bound drift-diffusion model (npb-DDM) to compute the corresponding time-dependent decision bound $B_{\theta_{\mathrm{initial}}}(t)$, which—together with *θ*_initial_—is used to evaluate the negative log-likelihood. The simplex algorithm then iteratively explores the parameter space, adjusting *θ* to minimize the objective function. To mitigate the risk of local minima, the procedure is repeated 10 times with different random initializations. We retain the parameter set that yields the highest likelihood across these runs (Table 1).

**Table 1.** Fitted model parameters for the npb-DDM

		Fitted parameters of the empirical bound model		
		*κ*	*μ*_nd_	*σ*_nd_
Participant	Phase	scaling factor for signal-to-noise	mean of non-decision time (s)	SD of non-decision time (s)
A	1	16.19	0.27	0.001
	2	20.53	0.30	0.036
	3	20.91	0.32	0.053
B	1	16.93	0.31	0.022
	2	16.26	0.27	0.027
	3	20.39	0.32	0.077
C	1	14.93	0.19	0.014
	2	16.24	0.25	0.001
	3	14.92	0.19	0.002
D	1	20.85	0.32	0.027
	2	22.89	0.28	0.035
	3	28.17	0.24	0.040

#### Model validation

We evaluated the ability of the npb-DDM to recover non-stationary decision bounds and model parameters by fitting the model to simulated data. For each of three bound dynamics—stationary, exponentially collapsing, and sinusoidally modulated—we generated 1,000 trials per motion coherence using a fixed set of parameters (*κ* = 15, *μ*_nd_ = 0.3, *σ*_nd_ = 0.01). The npb-DDM was then fit to each dataset, and the recovered bounds were compared to the true bounds used for simulation (Fig. 5*a*–*c*). To further assess parameter recovery, we simulated additional datasets using sinusoidally modulated bounds and a range of parameter combinations (Fig. 5*d*,*e*). Each dataset included 200 trials per coherence (comparable to the analyzed data in each phase). The values of *κ*, *μ*_nd_, and *σ*_nd_ were systematically varied, and we evaluated how accurately npb-DDM recovered both the bound shapes and the generative parameters. Accuracy was assessed by comparing the recovered parameters and bounds to their known ground-truth values.

The model successfully recovered the ground-truth bound shape and parameters, except for *σ*_nd_, which was slightly but systematically underestimated (Fig. 5*e*). This bias arises from (*i*) neglecting *σ*_nd_ when estimating the decision time distribution from RTs (see above) and (*ii*) the Epanechnikov kernel’s smoothing effect. Both factors increase the variance of the decision time distribution, which is compensated by a lower estimated variability in non-decision times. Importantly, this bias does not affect our capacity to identify the other parameters and the shape of the bounds, as the variability of the non-decision times is small compared to that of the decision times.

### Cross-fitting of scaled bounds

Here we describe the statistical analysis bearing on the claim that participants apply a scaled version of their unique bound shape across the phases of the experiment. For each participant and phase, we fit the npb-DDM to the data, $D_{i}^{Pk}$, where the subscript *i* ∈ {1…4} indexes the participants and the superscript *Pk* (*P*1 or *P*2) indicates experimental phases. This yields fit-parameters $\theta_{i}^{Pk}=[\kappa_{i}^{Pk},\mu_{nd,i}^{Pk},\sigma_{nd,i}^{Pk}]$, and bounds $B_{i}^{Pk}$. We ask how well the phase 2 data from participant *i* can be explained by a simple transformation of the bounds that were obtained from phase 1 data from participant *j*. The parameters of the DDM (signal-to-noise and non-decision time) were set to those obtained by fitting the npb-DDM to phase 2 data from participant *i* (i.e., $\theta_{i}^{P2}$). We use two free parameters, **s** = {*s*_t_, *s*_m_}, to “shrink” or “stretch” the bounds with respect to time and magnitude:

$$B^{\prime }(t)=s_{m}\cdot B_{\;j}^{P1}(t/s_{t}),\;(10)$$

and fit **s** using maximum log-likelihood:

$$\log\;P_{ij}=\max\left[\log\;P(D_{i}^{P2}|s,\theta_{i}^{P2},B_{j}^{P1})\right].\;(11)$$

The quality of the fit was quantified as the difference between the log-likelihood obtained from the best-fitting scaled-bound model and the npb-DDM:

$$\Delta \log\;P_{ij}=\log\;P_{ij}-\log\;P(D_{i}^{P2}|\theta_{i}^{P2},B_{i}^{P2}).\;(12)$$

If phase 2 data of participant *i* are best explained by scaling of phase 1 bounds from the same participant ( $B_{\;j}^{P1};j=i$) rather than from other participants (*j* ≠ *i*), then $\Delta \log\;P_{ij}$ should be greatest when *j* = *i*. Because of the different number of trials across the participants, we compared the average log-likelihood per trial (Fig. 8*b*): $\Delta \log\;L_{i,j}=(1/N_{i}^{P2})\Delta \log\;P_{i,j}$, where $N_{i}^{P2}$ is the number of trials for participant *i* in phase 2.

We used a permutation analysis to test the hypothesis that choices and RTs from phase 2 are best explained by scaling the decision bounds for phase 1 from the same participant, against the alternative that scaling from the same or different participants is inconsequential. For each participant *i*, we rank-ordered $\Delta \log\;P_{i*}$ and created a 4-by-4 matrix of the rank order. If each participant’s data were best explained by scaling his or her own bounds, then the diagonal elements of the matrix should be [1, 1, 1, 1], which sums to 4. In the data, the ranks were [1, 2, 1, 1], which sums to 5. There are four patterns (permutations) of these ranks that sum to 5. In contrast, under the null hypothesis, { $H_{0}$: the rank order is random}, there are 4^4^ ( = 256) possible patterns of the ranks across the diagonal elements. Therefore, the *p*-value that the sum of the ranks is 5 or less, as in our results, is (1+4)/256 (*p* = 0.020).

### Derivation of the optimal decision strategy

A decision policy maps a state of accumulated evidence and elapsed time to an action. The possible actions are to accumulate more information or to stop the decision process and make a left or right choice. Deriving the optimal decision policy is not trivial since it depends on the opportunity cost of time, which itself depends on the decision policy. We formulate the decision process as a Markov decision process (MDP) and use dynamic programming to derive the optimal policy (i.e., the one that maximizes points earned per unit of time) in each participant and phase of our experiment.

Our *Goal MDP* (Geffner and Bonet, 2013) comprises a non-empty state space *S*, an initial state *s*_0_, a goal state *s*_g_, a set of actions *A*(*s*) executable in state *s*, positive or negative reward *r*(*a*, *s*) obtained after performing action *a* in state *s*, and transition probabilities $P_{a}\left(s^{\prime }|s\right)$ representing the probability of transitioning to state $s^{\prime }$ after performing action *a* in state *s*.

The states *s* ∈ *S* are defined by a tuple *s* = 〈*x*, *t*〉, where *x* is the accumulated motion evidence in favor of the rightward target, and *t* is the elapsed decision time. At time zero, there is no accumulated evidence favoring either choice, *s*_0_ = 〈*x* = 0, *t* = 0〉. Three actions are applicable in each state: terminating the trial by making a left choice (left) or a right choice (right), or accumulating more evidence by maintaining the eye fixation (fix). Choosing a terminal action (left or right) leads to a cost-free absorbing goal state. In contrast, state transitions that follow the fixation (fix) are stochastic because of the noise in the momentary evidence even if the motion coherence was known. We discretize time and the amount of accumulated evidence in small steps of *δt* and *δx*, respectively.

The transition probability $p_{\mathrm{fix}}\left(s^{\prime }|s\right)$ specifies the likelihood of transitioning from state *s* = 〈*x*, *t*〉 to state $s=\langle x^{\prime },t^{\prime }\rangle$ after selecting action fix in state *s*. Because time is discretized in steps of *δt*, the only transitions with non-zero probability are those to states $s^{\prime }$ in which $t^{\prime }=t+\delta t$. The transition probabilities depend on motion coherence, which is unknown to the decision-maker. Thus, the optimal decision-maker must marginalize over the motion coherences to calculate $P_{\mathrm{fix}}\left(s^{\prime }|s\right)$ as follows:

$$p_{\mathrm{fix}}(s^{\prime }|s)=\sum_{i}p_{\mathrm{fix}}\left(s^{\prime }|s,{\mathrm{coh}}_{i}\right)\cdot p({\mathrm{coh}}_{i}|s),\;(13)$$

where $p_{\mathrm{fix}}\left(s^{\prime }|s,{\mathrm{coh}}_{i}\right)$ is the transition probability from state *s* to state $s^{\prime }$ if the motion coherence were ${\mathrm{coh}}_{i}$. As in the DDM, we assume that the evidence gathered in a small time step *δt* follows a normal distribution with a mean equal to $\kappa \cdot {\mathrm{coh}}_{i}\cdot \delta t$, and the variance equal to *δt*, thus

$$p_{\mathrm{fix}}(s^{\prime }|s,{\mathrm{coh}}_{i})=p_{\mathrm{fix}}\left(\langle x^{\prime },t+\delta t\rangle |\langle x,t\rangle ,{\mathrm{coh}}_{i}\right)=N(x^{\prime }-x|\kappa \cdot {\mathrm{coh}}_{i}\cdot \delta t,\delta t).\;(14)$$

The transition probability $P_{\mathrm{fix}}\left(s^{\prime }|s\right)$ also depends on $p({\mathrm{coh}}_{i}|s)$, the probability that motion coherence is ${\mathrm{coh}}_{i}$ when in state *s* = 〈*x*, *t*〉 (Eq. 13). This probability can be calculated by Bayes’ rule. Assuming that all motion coherences have equal prior probability (as in the experiment):

$$\begin{array}{rl} \;p\left({\mathrm{coh}}_{i}|\left\langle x,t\right\rangle \right) & =\frac{\;p\left(\left\langle x,t\right\rangle |{\mathrm{coh}}_{i}\right)\cdot p({\mathrm{coh}}_{i})}{\;p(\left\langle x,t\right\rangle )}\\ & =\frac{\;p\left(\left\langle x,t\right\rangle |{\mathrm{coh}}_{i}\right)\cdot p({\mathrm{coh}}_{i})}{\sum_{j}\;p\left(\left\langle x,t\right\rangle |{\mathrm{coh}}_{j}\right)\cdot p({\mathrm{coh}}_{j})}\\ & =\frac{\;p\left(\left\langle x,t\right\rangle |{\mathrm{coh}}_{i}\right)}{\sum_{j}\;p\left(\left\langle x,t\right\rangle |{\mathrm{coh}}_{j}\right)}. \end{array}\;(15)$$

Without decision-termination bounds,

$$p\left(\langle x,t\rangle |{\mathrm{coh}}_{i}\right)=N(x|\kappa \cdot {\mathrm{coh}}_{i}\cdot t,t),\;(16)$$

and thus

$$p({\mathrm{coh}}_{i}|\langle x,t\rangle )=\frac{1}{Z}N(x|\kappa \cdot {\mathrm{coh}}_{i}\cdot t,t),\;(17)$$

where *Z* is a normalization constant which assures that the sum of $p\left({\mathrm{coh}}_{i}|\langle x,t\rangle \right)$ over all coherences adds to one.

Following the approach of Drugowitsch et al. (2012), we show that Equation (17) still holds in the presence of termination bounds and provisional deadlines. Let $\bar{x}$ be a trajectory that leads to 〈*x*, *t*〉 and Ω(〈*x*, *t*〉) denote the set of all $\bar{x}$ leading to 〈*x*, *t*〉 without reaching the termination bounds. With termination bounds and provisional deadlines,

$$p\left(\langle x,t\rangle |{\mathrm{coh}}_{i}\right)=\left(1-p\left(T_{\mathrm{DL}}<t\right)\right)\cdot \int_{\Omega (\langle x,t\rangle )}p(\bar{x}|\kappa \cdot {\mathrm{coh}}_{i})d\bar{x},\;(18)$$

where *p*(*T*_DL_ < *t*) is the probability that a deadline occurs before time *t*, and $p(\bar{x}|\kappa \cdot {\mathrm{coh}}_{i})$ is the probability of a single trajectory $\bar{x}$, which can be expanded across time steps as follows:

$$\begin{array}{rl} \;p(\bar{x}|\kappa \cdot {\mathrm{coh}}_{i}) & =\left(\overset{t/\delta t-1}{\underset{n=0}{\Pi }}\;\frac{1}{\sqrt{2\pi \delta t}}\right)\cdot \exp\;\left[\sum_{n=0}^{t/\delta t-1}-\frac{1}{2}\cdot \frac{(\delta {\bar{x}}_{n}-\kappa \cdot {\mathrm{coh}}_{i}\cdot \delta t{)}^{2}}{\delta t}\right]\\ & =D(\bar{x})\cdot \exp\;\left[x(t)\cdot \kappa \cdot {\mathrm{coh}}_{i}-\frac{t}{2}\kappa^{2}\cdot {\mathrm{coh}}_{i}^{2}\right], \end{array}\;(19)$$

where $x(t)=\sum_{n=0}^{t/\delta t-1}\delta {\bar{x}}_{n}$ and $t=\sum_{n=0}^{t/\delta t-1}\delta t$ were used to substitute the sums. $D(\bar{x})$ is a function of the trajectory $\bar{x}$ and is independent of the motion coherence. That is, for each trajectory, there is a coherence-independent scaler $D(\bar{x})$, such that Equation (18) becomes

$$p\left(\langle x,t\rangle |{\mathrm{coh}}_{i}\right)=\left(1-p\left(T_{\mathrm{DL}}<t\right)\right)\cdot \int_{\Omega (\langle x,t\rangle )}D(\bar{x})d\bar{x}\cdot \exp\;\left[x(t)\cdot \kappa \cdot {\mathrm{coh}}_{i}-\frac{t}{2}\kappa^{2}\cdot {\mathrm{coh}}_{i}^{2}\right].\;(20)$$

In Equation (20), the coherence-dependent exponential term is multiplied by the coherence-independent terms that depends on the bound shape and the provisional deadline. Consequently, for any two motion coherences, ${\mathrm{coh}}_{i}$ and ${\mathrm{coh}}_{\;j}$, the ratio $p(\langle x,t\rangle |{\mathrm{coh}}_{i})/p(\langle x,t\rangle |{\mathrm{coh}}_{\;j})$ is independent of the bound shape and the presence of a provisional deadline, and equal to the ratio of two normal distributions indexed by Equation (16). Therefore, Equation (17) holds independently of the bound shape and the presence or absence of provisional deadlines.

To derive the policy that maximizes the points earning rate, we must take into account the opportunity cost associated with the passage of time. That is, while prolonging deliberation increases the decision accuracy on average, deliberating for too long will decrease the overall earning rate. Using established methods (Bertsekas, 1995), we find the decision policy that maximizes the earning rate. For phases 1 and 3 (no deadlines), the Bellman equation is

$$V(s)=\max\left\{ \begin{array}{rl} & Q(s,\mathrm{left})=\;p_{\mathrm{left}}(\mathrm{correct}|s)\cdot R_{c}+\;p_{\mathrm{left}}(\mathrm{error}|s)\\ & \;\cdot R_{e}-\rho \cdot (\mu_{\mathrm{ITI}}+\mu_{\mathrm{misc}}+\mu_{\mathrm{nd}}),\\ & Q(s,\mathrm{right})=\;p_{\mathrm{right}}(\mathrm{correct}|s)\cdot R_{c}+\;p_{\mathrm{right}}(\mathrm{error}|s)\\ & \;\cdot R_{e}-\rho \cdot (\mu_{\mathrm{ITI}}+\mu_{\mathrm{misc}}+\mu_{\mathrm{nd}}),\\ & Q(s,\mathrm{fix})=E[V(s^{\prime })|s]-\rho \cdot \delta t. \end{array}\right.\;(21)$$

*Q*(*s*, *a*) is the state-action value function representing the reward expected for performing action *a* in state *s*. *p*_*a*_(correct|*s*) is the probability of being correct after taking action *a* in state *s,* and *p*_*a*_(error|*s*) is calculated as 1 − *p*_*a*_(correct|*s*). Parameter *μ*_nd_ is the mean non-decision time, and *μ*_ITI_ is the mean inter-trial interval. *μ*_misc_ is a mean time for miscellaneous experimental latencies, including the time to acquire fixation, the delay after the fixation to the motion onset, and the validation time for a saccadic response. *R*_*c*_ and *R*_*e*_ are the rewards (points) obtained after a correct and error choice, respectively. The expectation in *Q*(*s*, fix) is an expectation over all future states $s^{\prime }$ that result from being in *s* and accumulate evidence for an additional *δt*. The earning rate, *ρ*, is unknown because it depends on the decision policy itself. Note that the subtraction of *ρ* · *μ*_·_ derives from recasting the Bellman equation in terms of maximizing average reward per unit time, as required by the differential form of the Bellman equation (Bertsekas, 1995). We searched for the value of *ρ* iteratively by root finding, using the bisection method (Bertsekas, 1995; Drugowitsch et al., 2012).

To derive the optimal policy for phase 2, we modified the equation for fixation by including the cost of trial cancelations due to deadlines:

$$\begin{array}{rl} Q(s,\mathrm{fix}) & =(1-p_{\mathrm{cancel}})\cdot (E[V(s^{\prime })|s]-\rho \delta t)+p_{\mathrm{cancel}}(s)\\ & \;\cdot (R_{\mathrm{cancel}}-\rho \cdot (\mu_{\mathrm{ITI}}+\mu_{\mathrm{misc}}^{\mathrm{cancel}}+\mu_{\mathrm{nd}})), \end{array}\;(22)$$

where *R*_cancel_ is the reward (points) following a trial cancelation. *p*_cancel_(*s*) is the probability that a deadline occurs at time *t* given that it has not occurred before. If all trials have a provisional deadline, then *p*_cancel_(*s*) is given by the hazard function of the Rayleigh distribution. However, because trials with and without provisional deadlines are randomly interleaved, *p*_cancel_(*s*) also depends on the probability that the trial has a provisional deadline (*d*^+^) given that time *t* has elapsed without a cancelation, *p*(*d*^+^|*t*):

$$p_{\mathrm{cancel}}(s)=\left\{ \begin{array}{cc} p(d^{+}|t)\cdot \delta t\cdot \frac{t-(t_{0}-\mu_{\mathrm{nd}})}{\sigma_{\mathrm{dl}}^{2}}\; & \text{if }t>(t_{0}-\mu_{\mathrm{nd}}),\\ 0 & \text{otherwise}. \end{array}\right.\;(23)$$

The probability that the trial has a deadline given that no deadline has been reached by time *t*, *p*(*d*^+^|*t*), can be calculated by Bayes’ rule:

$$\begin{array}{rl} \;p(d^{+}|t) & =\frac{\;p(t|d^{+})}{\;p(t)}\cdot p(d^{+})\\ & =\frac{\;p(t|d^{+})}{\;p(t|d^{+})\cdot P(d^{+})+p(t|d^{-})\cdot P(d^{-})}\cdot p(d^{+})\\ & =\frac{\;p(t|d^{+})}{\;p(t|d^{+})+p(t|d^{-})}, \end{array}\;(24)$$

where *p*(*t*|*d*^+^) and *p*(*t*|*d*^−^) are the probabilities that time *t* has elapsed without a cancelation on trials with and without a provisional deadline, respectively. *p*(*t*|*d*^−^) is equal to 1 because there are no cancelations due to provisional deadlines in *d*^−^ trials. *p*(*t*|*d*^+^) is given by the survival function for the Rayleigh distribution:

$$p(t|d^{+})=\exp\;\left[-\frac{1}{2}\cdot {\left(\frac{t-(t_{0}-\mu_{\mathrm{nd}})}{\sigma_{\mathrm{DL}}}\right)}^{2}\right].\;(25)$$

The optimal policies depicted in Figure 6 were derived with the following parameter values: *R*_*c*_ = +1 [point], *R*_*e*_ = −1 [point], *R*_cancel_ = 0 [point], *δt* = 0.5 [ms], *δx* = 0.02 [a.u.], *μ*_ITI_ = 2.0 [s], *μ*_misc_ = 0.7 [s], $\mu_{\mathrm{misc}}^{\mathrm{cancel}}$ = 0.4 [s] (note *μ*_misc_ is longer for non-canceled trials due to the saccade validation time), and fitted parameters (*κ* and *μ*_nd_) for each participant and phase (Table 1).

## Results

### A manipulation affecting the time cost of deliberation

Four human participants performed a RT version of the random-dot motion discrimination task (Fig. 1*a*). On each trial, the motion coherence was chosen at random from the set {±0, ±3.2, ±6.4, ±12.8, ±25.6, ±51.2%}, where the sign denotes the direction (negative for leftward and positive for rightward). We refer to the absolute value of coherence as the *motion strength*. When the participants reached decisions, they indicated their choice by making an eye movement to the right or left peripheral target. Participants were incentivized to make appropriate speed-accuracy tradeoff based on scoring of their performance. They earned one point for a correct choice and lost one point for an erroneous choice. The score did not change if they failed to maintain fixation or made an inaccurate eye movement to the target. When these events occurred, the ongoing trial stopped immediately and a next trial followed. After each trial, in addition to auditory feedback on the accuracy of the choice, they received visual feedback and a tally of the current point total as well as an intuitive graphic that displayed their points per minute (*earning rate*, from here on; Fig. 1*a*). Note that random guesses do not improve the earning rate because the expected score for a random choice is 0. Instead, they could increase the rate by answering quickly and accurately with saccades and by maintaining accurate gaze fixation, since inaccurate fixation would add time to the experiment, thereby lowering the earning rate.

The study was conducted in three phases (Fig. 1*b*). In phase 1, participants received extensive training until they achieved a stable earning rate. During training sessions, participants were acclimated to the game-like structure of the task. Each session lasted 13 min. Participants completed two sessions per day with a short break in between. They were specifically instructed to maximize their earning rate. We used the last 10 sessions (2,154–2,389 trials) to establish a baseline level of performance. In phase 2, unbeknownst to the participant, we imposed an additional time cost. On a random half of the trials, we chose a *provisional deadline*, *T*_DL_. If the participant deliberated beyond *T*_DL_, the trial was canceled with termination (Fig. 1*b*, yellow box). From the participant’s perspective, this occurrence was not dissimilar to other aborted trials, including those with an eye blink or an inaccurate fixation (Materials and Methods). *T*_DL_ was drawn from a Rayleigh distribution, bespoke to each participant. The parameters of the distribution were chosen to ensure that these provisional deadlines would come to fruition on approximately 10% of trials, absent a change in decision strategy (Fig. 1*c*). The other half of trials had no provisional deadline, and thus did not differ from trials in phase 1. In phase 3, we returned to the original paradigm without the deadlines. The phases were neither identified nor signaled to the participants. From their perspective, they were playing the same game for points.

**Figure 2.**
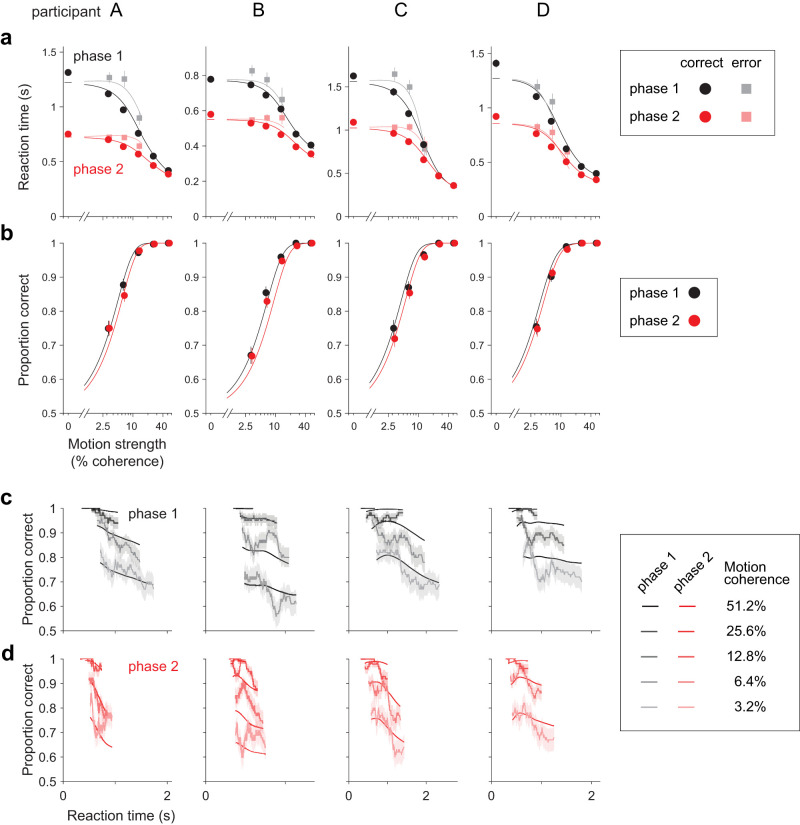
Effects of increased time cost on RT and accuracy for individual participants. ***a***, Mean RT for correct (circles) and error (squares) trials as a function of motion strength. Columns correspond to the four participants. Data from phases 1 and 2 are shown in black and red, respectively. Mean RTs for error trials are not shown if the participant had fewer than five error trials for a given motion strength. Error bars show SEM. The curves are fits of the npb-DDM (see Fig. 3 and Materials and Methods). ***b***, Proportions of correct choices as a function of motion strength for phase 1 (black) and phase 2 (red). Error bars show SEM. Curves represent the proportion of correct trials predicted by npb-DDM. ***c***, Proportion of correct choices as a function of RT, for trials from phase 1. The proportion correct was calculated independently for each motion strength, in sliding windows of 100 trials. Shading indicates SEM. The curves are fits of the npb-DDM. ***d***, As panel (***c***), for trials from phase 2. Trials with a provisional deadline were excluded from the data in all panels.

The manipulation in phase 2 affected task performance. All four participants reduced their RTs substantially, especially on the trials with low motion strength (Fig. 2*a*). For example, RTs decreased by 34.0 ± 7.5% at zero motion strength (mean ± SEM across four participants), while the decrease in RTs was only 8.6 ± 6.3% at the highest motion strength (Fig. 2*a*). The reduction in RT from phase 1 to phase 2 held for both correct and erroneous choices (dark and light symbols, *p* < 10^−37^ and *p* < 10^−20^, respectively, two-sample *t*-test on *z*-scored RT; see Materials and Methods). As we shall see, this suggests a change in the time-dependent criterion for terminating decisions. The increase in speed led the participants to beat the clock, so to speak. Instead of the 10% of trials that should have been terminated at *T*_DL_, less than 5% of trials were terminated by the provisional deadline (Table 2).

**Table 2.** Sampling distribution for provisional deadlines and the fraction of trials canceled or aborted

	Sampling distribution for provisional deadline				Canceled trials (%) in phase 2 (*n* = 20 sessions)	Aborted trials (%)		
Participant	*t*_0_ (s)	*σ*_DL_	Mean (s)	SD (s)		Phase 1 (*n* = 10 sessions)	Phase 2 (*n* = 20 sessions)	Phase 3 (*n* = 10 sessions)
A	0.6	0.7	1.48	0.46	1.4 ± 0.9	1.7 ± 1.4	2.2 ± 1.0	3.2 ± 1.2
B	0.3	0.6	1.05	0.39	4.3 ± 1.9	3.4 ± 1.2	2.5 ± 0.9	3.7 ± 2.0
C	0.6	1.0	1.85	0.66	3.6 ± 1.1	2.5 ± 1.3	2.7 ± 2.5	5.2 ± 2.9
D	0.6	0.6	1.35	0.39	3.7 ± 2.1	0.6 ± 0.5	1.3 ± 1.1	1.1 ± 0.9

The manipulation also affected decision accuracy. For each participant, accuracy was lower in phase 2 than in phase 1, even though we restrict the comparison to trials without provisional deadlines (Fig. 2*b*). We confirmed this qualitative observation by fitting a logistic regression model relating motion strength to choice accuracy (Eq. 3). The model, fit to phase 1 and phase 2 trials together, includes an indicator variable to identify phase 2 trials. For all participants, the regression coefficient associated with this indicator variable (*β*_Phase_; Eq. 3) is negative, indicating that accuracy was lower in phase 2. Although statistically reliable, the change in accuracy is subtle (Fig. 2*b*), achieving significance only in grouped data from the four participants ( $p=0.035;H_{0}\;:\;\beta_{\mathrm{Phase}}=0$; Eq. 4). Overall, the changes in behavior between phases 1 and 2 suggest a change in the tradeoff between decision speed and accuracy.

An explanation of the behavioral effects of the manipulation stems from the analysis of the time-dependent accuracy functions. For phase 1 trials with the same motion strength, faster decisions were, on average, more accurate (Fig. 2*c*). Such a decay in the time-dependent accuracy function may appear perplexing because slower decisions are thought to involve more deliberation, hence greater accuracy. What is missing from this intuition, however, is that the decision-maker is controlling the duration of the decision by exercising a stopping policy. The decrease in accuracy with slower decisions is a sign that the stopping criterion is more lax (i.e., that decision bounds *collapse* over time). The time-dependent accuracy functions in phase 2 showed a more pronounced decay than in phase 1 (Fig. 2*d*), suggesting that the behavioral adjustments in phase 2 may be mediated by lower and/or faster-collapsing bounds—an idea that we formalize below with a novel variant of the DDM.

### The speed-accuracy regime is controlled by the adjustment of the decision-termination bounds

**Figure 3.**
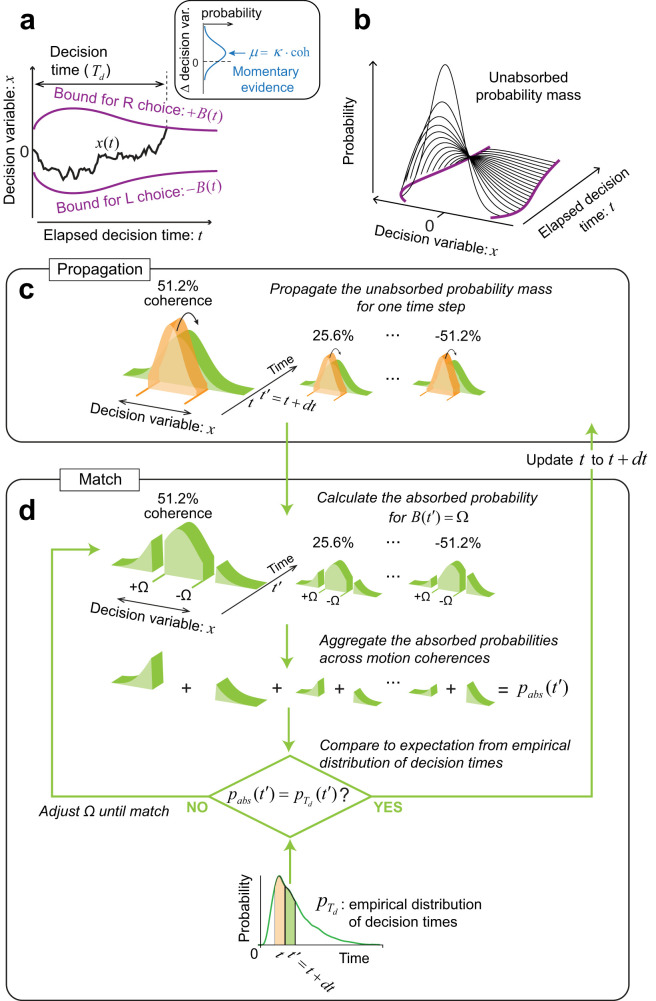
Bounded evidence-accumulation model. ***a***, Schematic representation of the DDM with time-dependent bounds. The DV, *x*(*t*), represents the accumulation of noisy momentary motion evidence over time. It follows a drift-diffusion process where the drift rate is proportional to the motion coherence (inset). The bounds are symmetric about the origin and vary as a function of time (purple curves). Reaching a bound determines the choice and decision time (*T*_d_) simultaneously. The observed RT is the sum of *T*_d_ and the non-decision time, *T*_nd_ (not shown). Only *T*_d_ depends on the direction and strength of motion. ***b***, If the decision bounds were known, the probability of terminating the decision at each moment in time (referred to as the *absorbed* probability) can be calculated iteratively. This involves propagating over time the probability of the state of *x*(*t*) (black curves) that remains unabsorbed by the bounds (purple curves) and measuring the probability mass that has been absorbed. ***c***, ***d***, Schematic illustration of the algorithm used to estimate the decision bounds, *B*(*t*). The algorithm proceeds iteratively over time, alternating between *propagation* of the state of the DV, *x*(*t*), and *match* to the absorbed probabilities inferred from the observed RTs. ***c***, The unabsorbed probability mass of the DV is propagated for one time step: from time *t* (orange) to *t*′ = *t* + *dt* (green). This step is performed independently for each motion coherence. ***d***, The *match* step establishes the height of the bound at time *t*′ that matches the probability of decision termination at time *t*′ between the model and the data. We calculate the absorbed probability for each motion coherence, assuming that the bound at time *t*′ is equal to Ω (top row of the panel). Absorbed probabilities are aggregated across motion coherences (second row). The total absorbed probability, *p*_abs_(*t*′), is compared to the value expected from the empirical distribution of RTs, $p_{T_{d}}(t^{\prime })$. We compute $p_{T_{d}}(t)$ from an estimate of decision times obtained by subtracting the expected non-decision time, *μ*_nd_, from each trial’s RT. We fit the resulting decision time estimates with a nonparametric kernel density function, from which we calculate $p_{T_{d}}(t)$ (bottom row). We find the value of Ω by root finding until *p*_abs_(*t*′) equals $p_{T_{d}}(t^{\prime })$. Then time is increased by *dt* and the process is repeated until all the probability mass has been absorbed.

To investigate which aspects of the decision policy changed from phase 1 to phase 2, we fit the data to a bounded evidence-accumulation model. In the model, the decision follows a drift-diffusion process with a constant diffusion coefficient and a drift rate proportional to the motion coherence (Fig. 3*a*). The decision process ends when the accumulated evidence (termed the decision variable, *DV*) crosses an upper or lower bound at ±*B*(*t*) signaling a rightward or leftward choice, respectively. The RT comprises the time it takes for the diffusing particle to reach one of the bounds (the decision time, *T*_d_) plus the non-decision time (*T*_nd_) that groups sensory and motor latencies, which are assumed independent of decision difficulty.

Given the parameters of a DDM, the probability of decision termination at any moment can be computed by propagating the unabsorbed states of the DV over time and recording the probability mass that is absorbed at the upper or lower bound (Fig. 3*b*). Our working hypothesis is that the changes in the speed-accuracy tradeoff from phase 1 to phase 2 are mainly explained by adjustments in the decision-termination bound, *B*(*t*).

**Figure 4.**
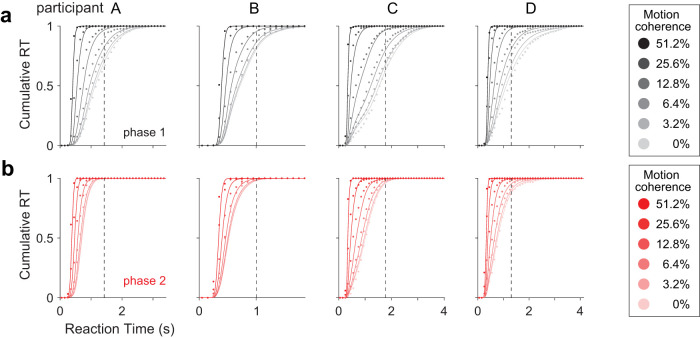
RT distribution across motion strengths predicted by *npb-DDM*. Cumulative distribution of observed RT (circles, 100 ms bins) and RT predicted by npb-DDM (curves) in phase 1 (***a***) and phase 2 (***b***). Trials with a provisional deadline were excluded from the data in phase 2. Vertical dashed lines show mean provisional cancelation time for each participant, calculated from phase 2 trials with provisional deadlines.

We therefore developed a method that allows us to derive the shape of the decision-termination bounds without assuming their functional form. We call this model the npb-DDM (Glickman et al., 2022). The key intuition behind this model is that for a given signal-to-noise, the distribution of decision times is uniquely determined by the shape of the decision bounds; we reverse the logic and use the distribution of empirical decision times to infer the shape of the time-varying decision bounds without fitting. Assuming that the variability of the non-decision times is small compared to that of the decision times (Zylberberg et al., 2016), the distribution of *decision times*, $p_{T_{d}}(t)$, can be approximated by the distribution of *RTs* after subtracting the mean non-decision time. The derivation of the bounds involves two steps, *propagation* and *match*. We assume that the state of the DV at time *t* = 0 is a delta function centered at *x* = 0 (no accumulated evidence for either choice). In the *propagation* step, the probability distribution for the state of the DV is propagated forward in time for a single time step, from *t* to *t* + *dt*. The propagation is performed independently for each motion coherence (Fig. 3*c*). In the *match* step, we determine where the bound should be placed at time *t*, such that the probability of crossing the upper or lower bound, when aggregated across motion coherences, is equal to the probability of making a decision at time *t* obtained from the empirical distribution of decision times, $p_{T_{d}}(t)$ (Fig. 3*d*). We iterate the *propagation* and *match* steps over time until all the probability mass has been absorbed at one of the decision bounds. The npb-DDM allows us to fit single-trial choice and RT data with only three parameters: a signal-to-noise parameter (*κ*) and two parameters for the distribution of non-decision times (see Materials and Methods). Despite its simplicity, the npb-DDM provides an excellent fit to RTs and accuracy for each phase and participant (Figs. 2, 4, solid lines).

**Figure 5.**
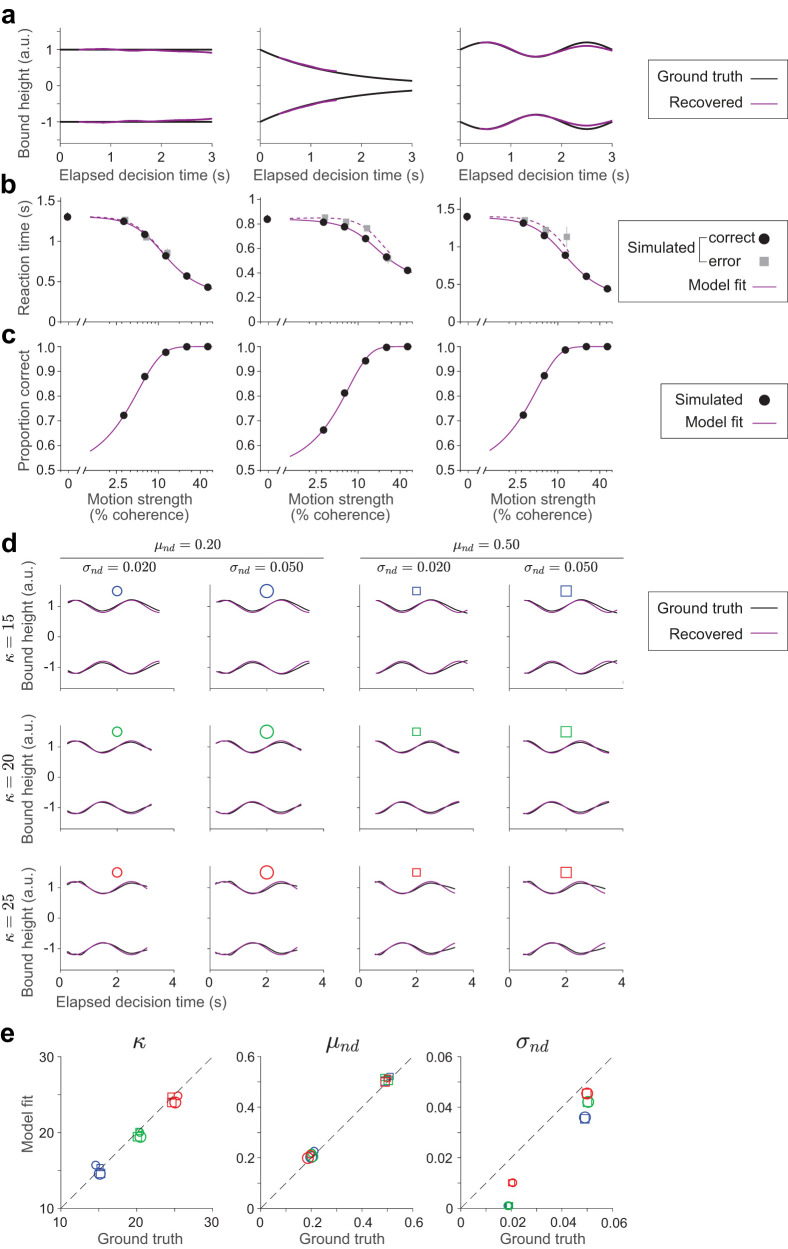
Validation of the *npb-DDM*. ***a***, We simulated 1,000 trials per motion coherence using a DDM with decision-termination bounds that are stationary (left), exponentially decaying (middle), or sinusoidally modulated (right) over time (black curves). The same set of model parameters was used for all simulations (*κ* = 15, *μ*_nd_ = 0.3, *σ*_nd_ = 0.01). Purple curves show the decision bounds recovered by npb-DDM, plotted over the time window spanning the 1st to 99th percentile of decision times. ***b***, Simulated RTs for correct and error trials (circles and squares, respectively) as a function of motion strength. Error bars show mean ± SEM. Purple curves are fits of npb-DDM. ***c***, Simulated proportion correct as a function of motion strength (circles). Purple curves are fits of npb-DDM. ***d***, Bounds recovered by npb-DDM by fitting data simulated for 200 trials per motion coherence with sinusoidally modulated bounds and various parameter sets. Symbols represent different parameter sets, varying in *κ* (color), *μ*_nd_ (shape), and *σ*_nd_ (size). The bounds are shown for the time window spanning the 1st to 99th percentile of decision times. ***e***, Model parameters recovered by npb-DDM from the data simulated in panel (***d***). The same symbols as in panel (***d***) denote the model parameters used for simulation (ground truth) and those recovered by npb-DDM (model fit).

To evaluate the reliability of the npb-DDM in estimating the model parameters, we fit the model to synthetic data. Our fitting procedure successfully recovered the ground-truth bound shape and parameters (Fig. 5), except for a slight but systematic underestimation of *σ*_nd_ (Fig. 5*e*; see Materials and Methods for details). Importantly, because non-decision time variability contributes only a small fraction of the total RT variability in the task (Zylberberg et al., 2016), this underestimation has minimal impact on the model’s overall performance.

### Bound adjustments are consistent with earning rate maximization

**Figure 6.**
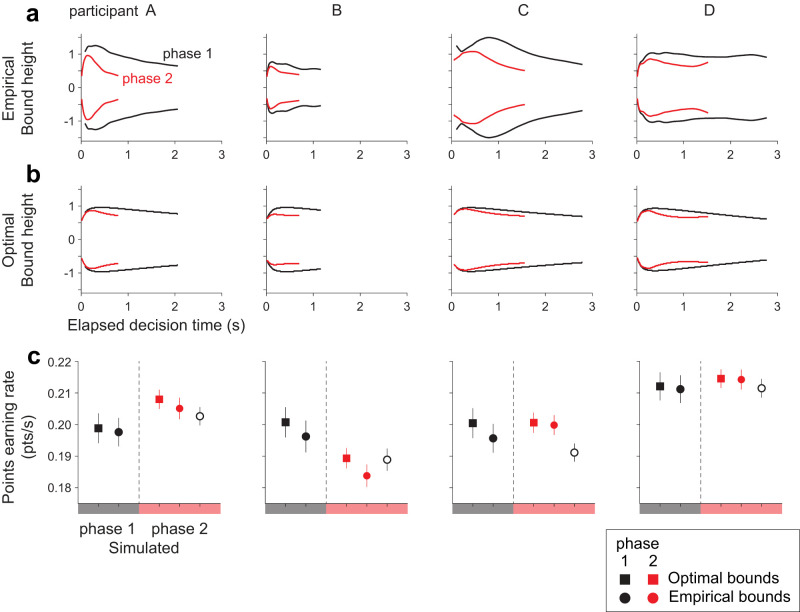
Adjusted decision-termination bounds under increased time costs. ***a***, Shape of the decision-termination bounds estimated with the npb-DDM. Bound shapes for phases 1 and 2 are shown in black and red, respectively. The bound shapes were derived from observed RTs (Fig. 3). The bounds are shown for the range corresponding to the 1st–99th percentile of decision times for each participant and phase. Columns correspond to the four participants. ***b***, Shape of the decision-termination bounds that would maximize the points earning rate. Optimal bounds for phase 1 and phase 2 are shown in black and red, respectively. The bound shapes were derived using dynamic programming to solve the Bellman equations. Signal-to-noise (*κ*) and mean non-decision time (*μ*_nd_) were fixed at the values obtained from the npb-DDM fits for each participant. ***c***, Simulated points earning rate in phase 1 and phase 2 with optimal and fitted bounds. Symbols and error bars show mean  ± SEM of points earning rate in each phase (10 sessions in phase 1 or 20 sessions in phase 2). Open symbols show the expected points earning rate if the decision-termination bounds from phase 1 were used in phase 2 without adjustment.

**Figure 7.**
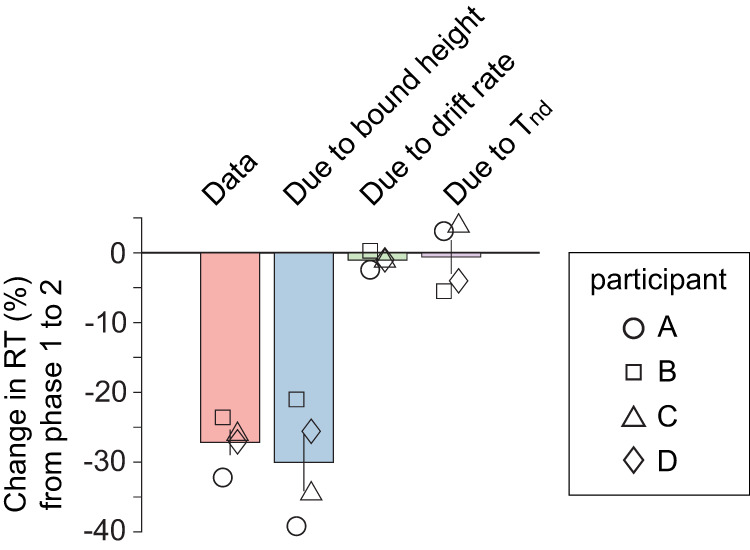
RT reduction due to the change in each model parameter from phase 1 to phase 2. Reduction in mean RT (%) from phase 1 to phase 2. Error bars show mean across participants. Individual participant data are indicated by the open symbols. The leftmost (red) bar corresponds to the experimentally observed change in RT from phase 1 to phase 2. The next three bars correspond to model expectations. These are obtained from numerical solutions of the npb-DDM with parameters fit to the phase 1 data, except for one of the parameters that is replaced with the value obtained from fitting phase 2 data. The replaced parameters are bound height (blue bar), drift rate (green), and mean non-decision time (purple). The reductions in mean RT are significantly different from 0% for the changes due to bound height (*p* = 0.0027; one-tailed *t*-test), but not for the changes due to drift rate (*p* = 0.078) or *T*_nd_ (*p* = 0.41).

The fitted models show that the participants decreased their decision-stopping criterion, *B*(*t*), to accommodate the increased time cost in phase 2. Figure 6*a* shows the shape of the bounds inferred by the model for phases 1 (black) and phase 2 (red). For all participants, the bounds were closer to zero in phase 2 than in phase 1. The change in bounds accounts for almost all of the RT reduction from phase 1 to phase 2, while changes in other model components (drift rate or non-decision time) account for only a small reduction (Fig. 7). The lowered bound height primarily reduced the proportion of trials with long RTs, explaining a more pronounced reduction in the RT for lower coherence trials (Fig. 2*a*). In contrast, a decrease in the decision accuracy was moderate for lower coherence trials because the accuracy in low coherence trials was close to, or at, chance level even during phase 1 (Fig. 2*b*). Therefore, the pattern of bound adjustment from phase 1 to phase 2 is sensible as it speeds up what would have been low-accuracy long-RT trials.

We used dynamic programming to determine whether the adjustment of the bounds approximates the adjustment expected under the optimal policy. In this framework, a decision policy is a deterministic mapping from a state of accumulated evidence and time to an action. The optimal decision policy can be succinctly represented by bounds that separate regions of the state space where it is favorable to continue gathering evidence from those regions of the state space where it is favorable to make a rightward/leftward choice. The optimal policy was derived independently for each participant and each phase of the task, using the parameters obtained from the fits of the npb-DDM. The optimal strategy is to set the bounds closer to zero in phase 2 (Fig. 6*b*), similar to what we obtained from the fits of the npb-DDM (Fig. 6*a*). Based on our simulation (Fig. 6*c*), three out of four participants (A, C, and D) would have experienced a lower earning rate had they carried their bounds from phase 1 to 2 without adjustment (filled red circle vs. open black circle in Fig. 6*c*). Overall, the participants achieved 99% of the optimal points earning rate in each phase (99.2 ± 0.7% in phase 1, 99.2 ± 0.7% in phase 2, *n* = 4 participants; Fig. 6*c*), supporting the notion that they sensibly adapted the decision bounds to the time cost manipulation.

### Bound shape is idiosyncratic

**Figure 8.**
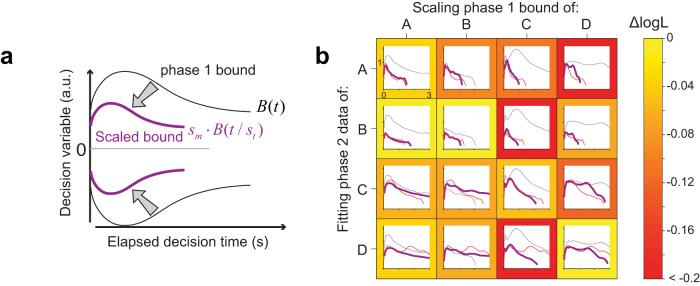
Scaling of phase 1 decision bound to fit phase 2 data. ***a***, Schematic illustration of the transformation method. The bounds obtained from phase 1 (black curves) are scaled linearly as a function of magnitude and time to fit the data from phase 2 (only trials without a provisional deadline). The scaling parameters (*s*_t_ and *s*_m_) were fit to the RT and choice data from phase 2. ***b***, Distinct bound profiles associated with individual participants. The grid shows the degree to which the phase 1 decision bound (black curve) for participant *i* (column) can be transformed to explain the phase 2 data for participant *j* (row). The thick purple curve indicates the best-fitting decision bounds obtained with the bound-scaling approach. The red curve shows the decision bounds derived from fitting the npb-DDM to the phase 2 data for participant *j* (row). Note that the purple and red curves tend to be more similar when the same participant’s data are used for scaling and fitting (grids along the diagonal). The frame color of each grid reflects the quality of the fit, quantified as the difference in log-likelihood per trial ( ΔlogL; color bar) between the model that uses the bound-scaling approach and the npb-DDM fit to phase 2.

The shape of the decision bounds derived from the npb-DDM fits do not seem to have a simple parameterization (e.g., constant or exponential; Fig. 6*a*). Therefore, it is unclear how many degrees of freedom (parameters) the brain may need to control in order to establish the decision bound. More parameters would provide more flexibility, but at a cost—that is, it would take many trials to find the right combination. An examination of the bound shapes suggests that each participant established a unique, idiosyncratic bound during phase 1, and scaled their bespoke shape to accommodate the cost of time in phase 2. To evaluate this inference, we used the bound shape obtained from phase 1 of the task to fit the data of phase 2. We allowed the bounds obtained from phase 1 to change by simple multiplicative scaling in time and/or magnitude (Fig. 8*a*). This procedure was performed using each participant’s data in phase 2 or each of the other participants’ data in phase 2 (Fig. 8*b*). The scaled bounds tended to explain the behavior of the same participant better than those of the others (*p* = 0.02, see Materials and Methods). While this result should be considered preliminary given the small number of participants, it suggests that the termination criterion of a decision (and hence, also the distribution of RTs and confidence judgments) may be an individual trait (Ais et al., 2016).

### Rapid adjustment of decision policy after experiencing a cancelation

**Figure 9.**
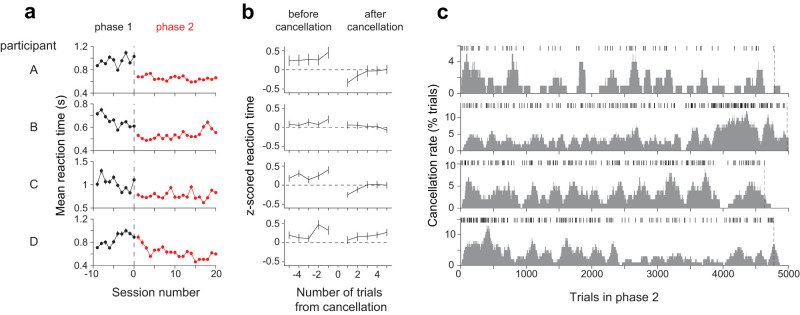
Effect of canceled trials on RT. ***a***, Mean RT across sessions in phase 1 (black circles) and phase 2 (red circles). Trials with provisional deadlines were excluded from the analysis. Error bars show SEM. ***b***, Mean RT before and after a canceled trial. Rows correspond to the four participants. RTs from phase 2 are *z*-scored independently for each motion strength, and the mean *z*-scored RTs are shown for five trials before and after the trial cancelation. Error bars show SEM. Note that the mean *z*-scored RTs tend to be positive before the cancelation. This is a sign that cancelations are more likely to occur when participants are responding slower than average. ***c***, Distribution of trial cancelations in phase 2. Rows correspond to the four participants. The ticks indicate trials in which the deadline was reached, resulting in cancelation. The trial cancelation rate (gray area) was calculated using a sliding window of 100 trials and plotted at the last trial in the window (rightmost edge of the window). Vertical dashed lines mark the last trial of phase 2. Panels ***b*** and ***c*** include all trials in phase 2 (with and without a provisional deadline).

Our task requires adjusting the decision policy over time based on scattered unpredicted signals—that is, the trial cancelations. These adjustments occurred despite limited exposure to trial cancelations, as three out of four participants showed a marked increase in decision speed within the first session of phase 2 (Fig. 9*a*; *p* < 0.001 for participants A, B, and C, *t*-test). During this session, their mean RT decreased by 15–40%, even though they encountered only 9–12 canceled trials. This sudden change could not be solely attributed to a gradual reduction in RT across sessions (*p* < 10^−5^ for all subjects, linear regression, see Materials and Methods). To analyze how canceled trials affected subsequent decisions, we compared the RTs before and after canceled trials. We combined trials across different coherences by standardizing (*z*-scoring) the RT separately for each coherence. Standardized RTs were reduced immediately after a trial cancelation (Fig. 9*b*; *p* < 0.01 for participants A, C, and D, paired *t*-test comparing RTs on the last two trials before and the first two trials after a canceled trial), and increased gradually as participants experienced a sequence of trials without cancelations (Fig. 9*b*), which explains why trial cancelations were broadly distributed across sessions and not just limited to the first few sessions of phase 2 (Fig. 9*c*). These results suggest that the participants adjusted their bound height to the environmental statistics throughout the experiment.

**Figure 10.**
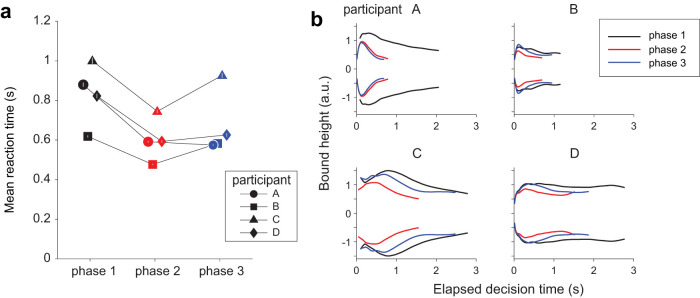
Partial recovery of phase 1 bounds after elimination of provisional deadlines. ***a***, Mean RT across phases, for each participant (symbols). Trials with a provisional deadline were excluded from the data in phase 2. Symbols and error bars show mean ± SEM. Color in both panels indicates experimental phase. ***b***, Shape of the decision-termination bounds derived with the npb-DDM for phases 1–3. The bounds are shown for the range corresponding to the 1st–99th percentile of decision times for each participant.

In the final phase 3 of the task, we removed the provisional deadlines to reduce the time cost. Three participants slowed their decisions speed (*p* < 0.01 for participants B, C, and D; Fig. 10*a*), achieving a speed and accuracy that was somewhere between those of phases 1 and 2. They did so by increasing the height of the decision-termination bounds relative to those adopted in phase 2 (Fig. 10*b*). However, the height of the bound did not quite reach that of phase 1, even though the experimental design was identical across these two phases. We can only speculate that they still carried a belief, albeit non-conscious, that time can be costly, or perhaps they incorporated the possibility of change in the environment (e.g., volatility). These interpretations aside, the result clearly attests to the flexibility of the time-dependent stopping policy in decision making.

## Discussion

We have shown that human observers exercise flexible and refined control over the policy to terminate decisions based on accumulated evidence. For decisions based on a sequence of independent samples of noisy evidence, it is sensible to deliberate until a sufficient quantity of information has accrued. The process may be formalized as a type of sequential sampling of evidence with optional stopping, and with suitable simplifications, it is analogous to bounded drift-diffusion or a biased random walk to bound (Ratcliff, 1978; Smith, 1990; Bogacz et al., 2006; Bogacz, 2007). The bounded DDM in particular offers a parsimonious explanation of both decision accuracy and speed.

Perceptual decisions about the direction of dynamic random-dot motion invite the simplifications of the DDM because, on any single trial, samples of evidence are statistically stationary and effectively independent. In many instances, it might make sense to estimate difficulty before deliberating about the question at hand, but owing to the high concentration of difficult conditions in our task, assessing difficulty (i.e., motion strength) would not be fruitful. Specifically, it would not be fruitful to aim for some level of accuracy based on an assessment of motion strength. Indeed participants appear to apply a common criterion to all motion strengths, which leads to the graded accuracy functions in Figure 2*b*.

The termination rule is an expression of policy in both a mathematical and pragmatic sense. The mathematical sense of policy is borrowed from the field of dynamic programming. By iterating over possible stopping rules, one settles on symmetric bounds, ±*B*(*t*), that maximize desiderata. In our experiment, per instruction, this should have been the points earning rate. By pragmatic, we mean that the termination rule is the only important control that the participant can deploy to perform this task. The reliability of the evidence (i.e., signal-to-noise ratio) is controlled by the experimenter and the response of direction-selective neurons in the visual cortex (Shadlen and Newsome, 1994, 1998; Born and Bradley, 2005). A central question in neuroscience concerns the mechanisms responsible for establishing and implementing this bound. Our study contributes to our understanding of the former. It shows that terminating bounds, which operate on the level of accumulated evidence, are dynamic and adjustable, and it shows that they can be adjusted to approximate optimal performance. The latter mechanism of termination has been shown to involve the superior colliculus (Stine et al., 2023).

The tradeoff between speed and accuracy is mediated by control of the threshold, or bounds, for terminating a decision. In the standard DDM, these bounds are assumed to be stationary, that is, time-independent or flat. However, this simplification, inspired by Wald’s sequential probability ratio test (Wald, 1947; Wald and Wolfowitz, 1948), would also predict that the time-dependent accuracy functions—for any given motion strength—should also be flat. Clearly they are not flat, but decline as a function of RT (Fig. 2*c*,*d*). Such collapsing bounds also explain the longer RTs accompanying errors (Fig. 2*a*) and the shapes of RT distributions (Fig. 4). Indeed, collapsing bounds are the natural extension of Wald’s normative theory for the common situation in which a variety of possible difficulties might be encountered (Drugowitsch et al., 2012). The key intuition is that as time elapses, a decision that has yet to terminate is more likely to be based on evidence from a less reliable source (i.e., lower signal-to-noise) and hence more likely to result in an error. Consequently, the deliberation time is progressively more costly. Collapsing bounds might also incorporate other costs associated with deliberation time, such as mental effort (e.g., allocation of attentional resources).

The manipulation introduced in phase 2 of our experiment provides a direct test of these ideas. It shows that the bounds change shape in response to a subtle change in experimental conditions. We argue that the shape of the bounds in phases 1 and 3 of the experiment also reflect the cost of decision time, but we did not test this directly, for example, by changing the point system. Our manipulation led to shorter RTs, and they did so in a way that is most consistent with linear scaling of the bounds. The essential feature of this mechanism is that decisions terminate based on the state of the evidence.

An alternative to a change in bounds is that the intervention applied in phase 2 made participants more attentive. This could speed up decisions if a change in attention were to reduce noise of the sensory representation, as is commonly believed (McAdams and Maunsell, 1999; Bichot et al., 2005; Cohen and Maunsell, 2009). However, this mechanism would not explain the change in RT at the lowest coherences. In fact, lowering the noise at the lowest coherences would increase RT because those decision times are dominated by diffusion, not drift (Zylberberg et al., 2016). Supporting this interpretation, we found that the signal-to-noise parameter (*κ*) explained only a small fraction of the reduction in RT from phase 1 to phase 2 (Fig. 7).

The mechanism at play in all three phases of the experiment helps the participant maximize their points earning rate. We estimated this maximum earning rate using dynamic programming. After an adjustment period, participants came within 99% of the optimal rate. We are loath to read too much into this small discrepancy. The process is, after all, confounded by perceptual learning and other adjustments (e.g., to the eye tracker). Moreover, the estimate of optimal performance assumes perfect knowledge of the prior over stimulus-difficulty and the temporal structure of the task, whereas participants can only approximate these factors. Finally, although the definition of optimal is clear, it is hard to know what participants actually experience. For example, it has been argued that mental effort is itself costly (Drugowitsch et al., 2012).

Our nonparametric method for finding decision bounds allowed us to identify features of their shape that could have been overlooked by more conventional methods based on parametric forms. Using the npb-DDM, we found that the shape of the bounds was highly consistent across different phases of the task. This consistency was revealed by an analysis in which we used simple transformations—linear scaling in time and magnitude—applied to the bounds obtained from phase 1 to explain the data of phase 2. The behavioral data in phase 2 was best explained by scaling the bounds obtained from the same (rather than different) participant. This result suggests that bound shape is idiosyncratic. This idiosyncrasy is not expected from the solution of the optimal model, which produced similar bounds across participants. However, as previously noted, this result should be interpreted with caution given the limited number of participants.

People (and other animals) often take longer to make a choice when the decision is preceded by another one in which they chose incorrectly, a phenomenon known as post-error slowing (PES; Rabbitt, 1966). This increase in RT is due to an increase in the amount of evidence required to make a choice, mediated by a change in the height of the decision-termination bounds (Purcell and Kiani, 2016). In our experiment, the cancelation of a trial also led to an adjustment in response speed, but in the opposite direction to that observed in the PES. That is, people were faster immediately after experiencing a cancelation (Fig. 9*b*). This distinction suggests that the adjustment of decision policy after an error requires a causal inference about the origin of the error: insufficient urgency versus a lax stopping criterion. We imagine that the degree of confidence in the evolving but canceled decision might inform this assignment, but this remains to be seen. At the very least, the distinction attests to the flexibility of the decision-making process, and the possibility of precisely and rapidly altering the parameters that control the speed-accuracy regime.

Our study does not address the neural mechanisms underlying the establishment and implementation of the bound, but studies in non-human primates provide insight. When monkeys perform the direction discrimination task, they also exhibit the same regularities as our human participants, including declining time-dependent accuracy, slow errors, and RT distributions suggestive of collapsing decision bounds (Drugowitsch et al., 2012; Kira et al., 2015). However, the neural implementation is not a change in a threshold per se. Neurons in several brain areas are known to represent the accumulation of noisy evidence resembling an integral of the difference in signals from left- and right-preferring direction-selective neurons in middle temporal and middle superior temporal areas. The activity of neurons in the lateral intralparietal area (LIP) gives a clear indication of a terminating threshold—a point of minimum variance just before the response (Roitman and Shadlen, 2002; Churchland et al., 2011; Kira et al., 2015)—implying that a downstream area terminates the decision when the firing rate reaches a critical level. However, this threshold is not time-dependent. Instead, the DDM is realized by a race between two accumulators: one accumulating evidence for leftward (and against rightward) motion and the other accumulating evidence for rightward (and against leftward) motion (Mazurek et al., 2003; Gold and Shadlen, 2007; Kira et al., 2015; Zylberberg and Shadlen, 2025); the first of these accumulators to reach a positive termination bound determines the choice. In this architecture, a change in bound height is equivalent to a change in starting point for both processes, and a collapsing bound can be implemented as a time-dependent signal that is added to both accumulators, what we term an urgency signal. This urgency signal is evident in the neural responses in area LIP (Churchland et al., 2008; Kira et al., 2015) and it changes if the monkey alters the tradeoff between speed and accuracy (Hanks et al., 2014).

The implication is that setting *B*(*t*) and detecting a bound crossing are likely to involve different structures. The first involves the generation of a time-dependent urgency signal that is added to the neural representation of cumulative noise and signal (Janssen and Shadlen, 2005; Churchland et al., 2008; Hanks et al., 2014; Kira et al., 2015), whereas the latter is a simpler threshold crossing detection that operates on the firing rates of these neurons. It can be carried out by a downstream structure, such as the superior colliculus, in a manner that is consistent regardless of time costs (Stine et al., 2023). There is something to be said for the parsimony of this design. Whereas the establishment of *B*(*t*) appears to accommodate detailed policy considerations, the termination threshold appears to operate generically, that is to say, independent of these considerations.
